# Supplementary material for: Breast cancer remodels lymphatics in sentinel lymph nodes
Source: Nat Commun. 2025 Nov 17;16:10056. doi: 10.1038/s41467-025-64981-z (PMC12623973; doi:10.1038/s41467-025-64981-z)
Supplement: Supplementary file 1 — Supplementary Information [file 41467_2025_64981_MOESM1_ESM.pdf]

a

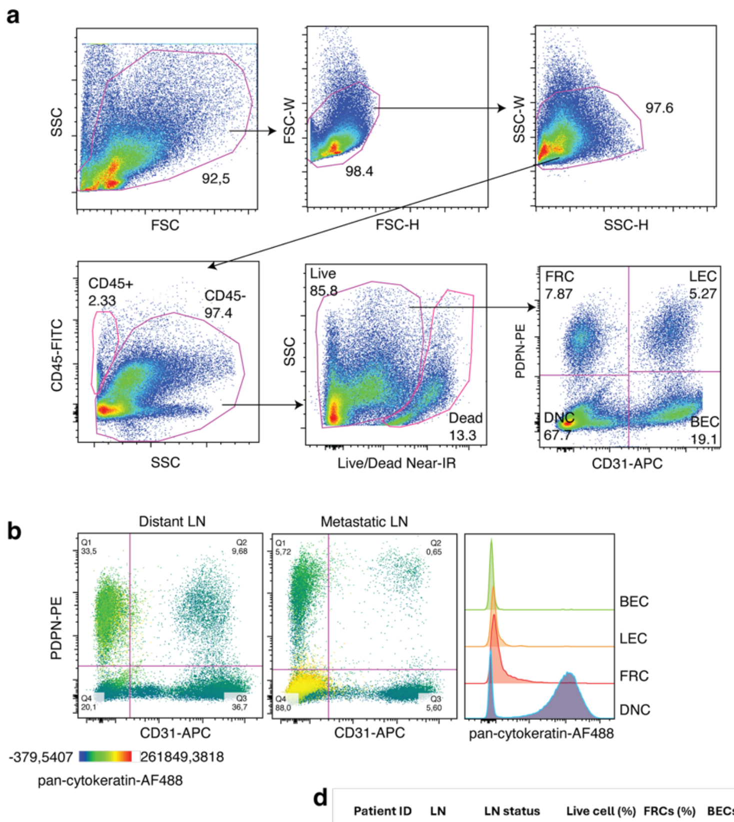

c

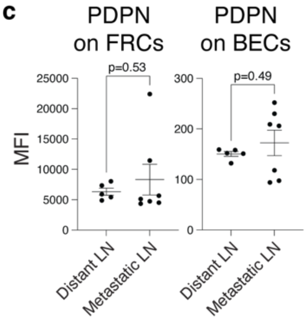

d

Patient ID	LN	LN status	Live cell (%)	FRCs (%)	BECs (%)	LECs (%)
#1	AXLN1	Metastatic	89.3	0.96	11.3	2.23
#1	AXLN2	Metastatic	85.8	2.92	14	5.06
#1	AXLN3	Metastatic	89.7	1.01	14.8	2.5
#1	AXLN4	Metastatic	90.9	1.57	11.6	4.77
#2	AXLN1	Metastatic	68.7	17.6	15.3	2.89
#3	AXLN1	Metastatic	67.5	12.9	34.6	4.41
#3	AXLN2	Non-metastatic	86.9	10.9	25.9	7.26
#4	AXLN1	Metastatic	71.8	5.45	21	5.71
#4	AXLN2	Non-metastatic	90.7	9.46	38.2	5.62
#5	AXLN1	Metastatic	83.4	1.86	2.26	0.33
#5	AXLN2	Non-metastatic	84.2	16	24.9	3.76
#5	AXLN3	Non-metastatic	79.2	16.1	18.1	3.68
#5	AXLN4	Metastatic	79.8	7.59	2.49	0.77
#6	AXLN1	Metastatic	83.6	1.94	3.82	1.95
#6	AXLN2	Metastatic	87.5	5.44	1.8	1.34
#6	AXLN3	Non-metastatic	93.9	3.04	2.44	1.43
#7	AXLN1	Metastatic	87.8	24.5	22.2	2.81
#7	AXLN2	Non-metastatic	94.7	16.8	44	10
#8	AXLN1	Metastatic	93.5	9.11	21.1	4.17
#8	AXLN2	Non-metastatic	87.4	15	29	6
#9	AXLN1	Metastatic	66.5	60.9	15.3	1.21
#9	AXLN2	Non-metastatic	77	9.12	41.9	10.7

Supplementary Fig. 1 | LEC Isolation. a Gating strategy for LEC enrichment. Doublets were excluded, followed by gating of $CD45^-$ cells. Among Live/Dead Near-IR-negative live cells, $PDPN^+$ $CD31^+$ double-positive cells were selected and sorted. This gating strategy corresponds to the data shown in Fig. 1a of the main manuscript. **b** $PDPN^-CD31^-$ double-negative cells (DNCs) include cancer cells. Dot plots (left) display the intensity of pan-cytokeratin expression (indicated by color). Histograms (right) show pan-cytokeratin expression in FRCs ($PDPN^+CD31^-$), LECs ($PDPN^+CD31^+$), BECs ($PDPN^-CD31^+$), and DNCs ($PDPN^-CD31^-$) cells. **c** PDPN expression in FRCs and BECs from distant (n=5 patients) and metastatic LNs (n=7 patients) (mean±SEM, two-tailed, unpaired Student's t-test). Source data are provided as a Source Data file. **d** Frequencies of live cells within the $CD45^-$ fraction and the proportions of FRCs, BECs, and LECs among live cells across all samples used in this scRNA-seq study.

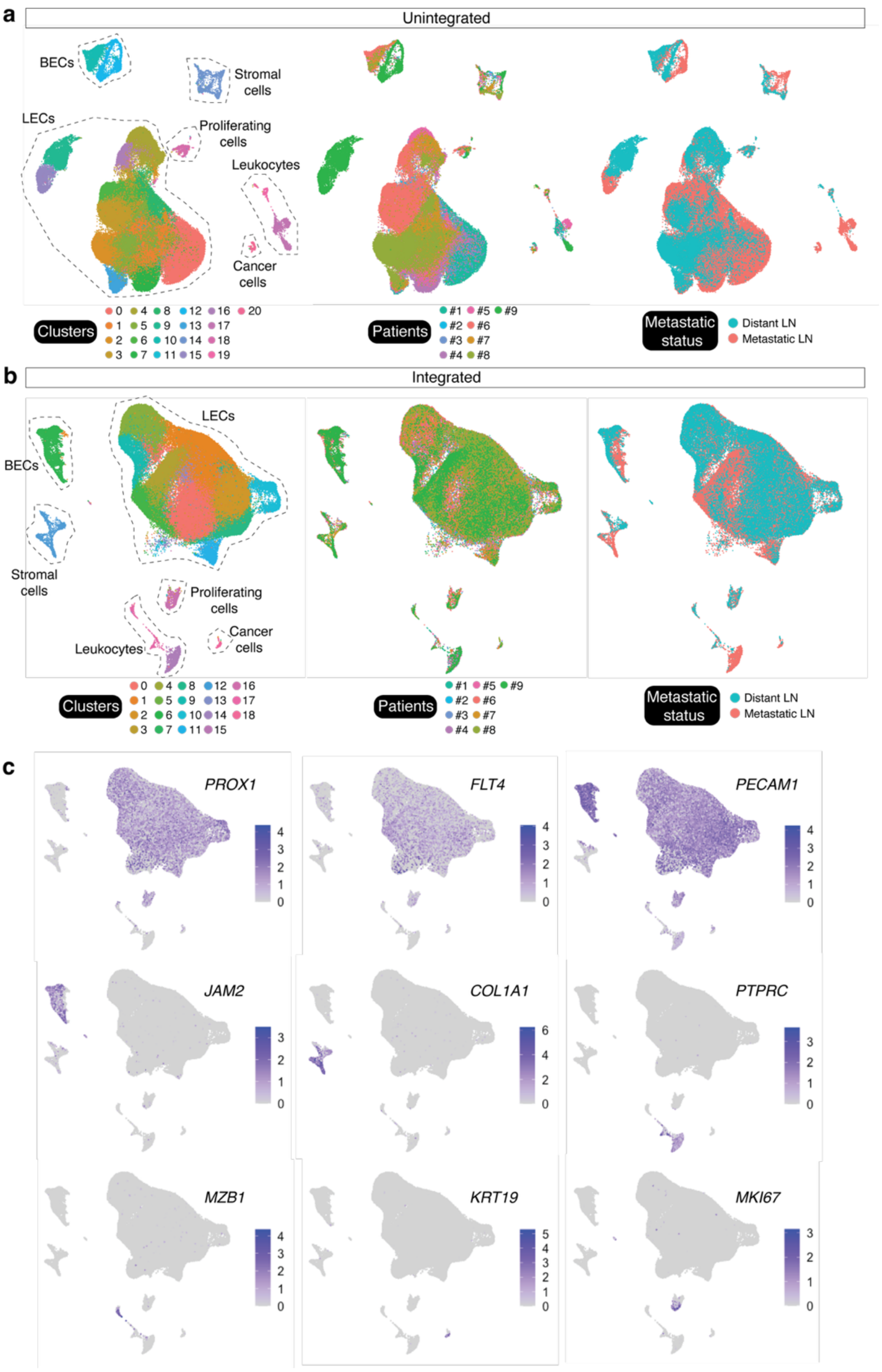

Supplementary Fig. 2 | Cell subsets in LEC-enriched populations from metastatic and distant LNs. a, b UMAP plots of unintegrated (a) and integrated (b) LEC-enriched populations from distant and metastatic human LNs from 9 patient samples. Plots are colored by clusters (left), patients (middle), and metastatic state (right). **c** Feature plots showing markers for each population. Markers include *PROX1* and *FLT4* for LECs, *JAM2* for BECs, *PECAM1* for ECs, *COL1A1* for stromal cells, *PTPRC* (CD45) for leukocytes, *MZB1* for plasmablasts, *KRT19* for cancer cells, and *MKI67* for proliferating cells.

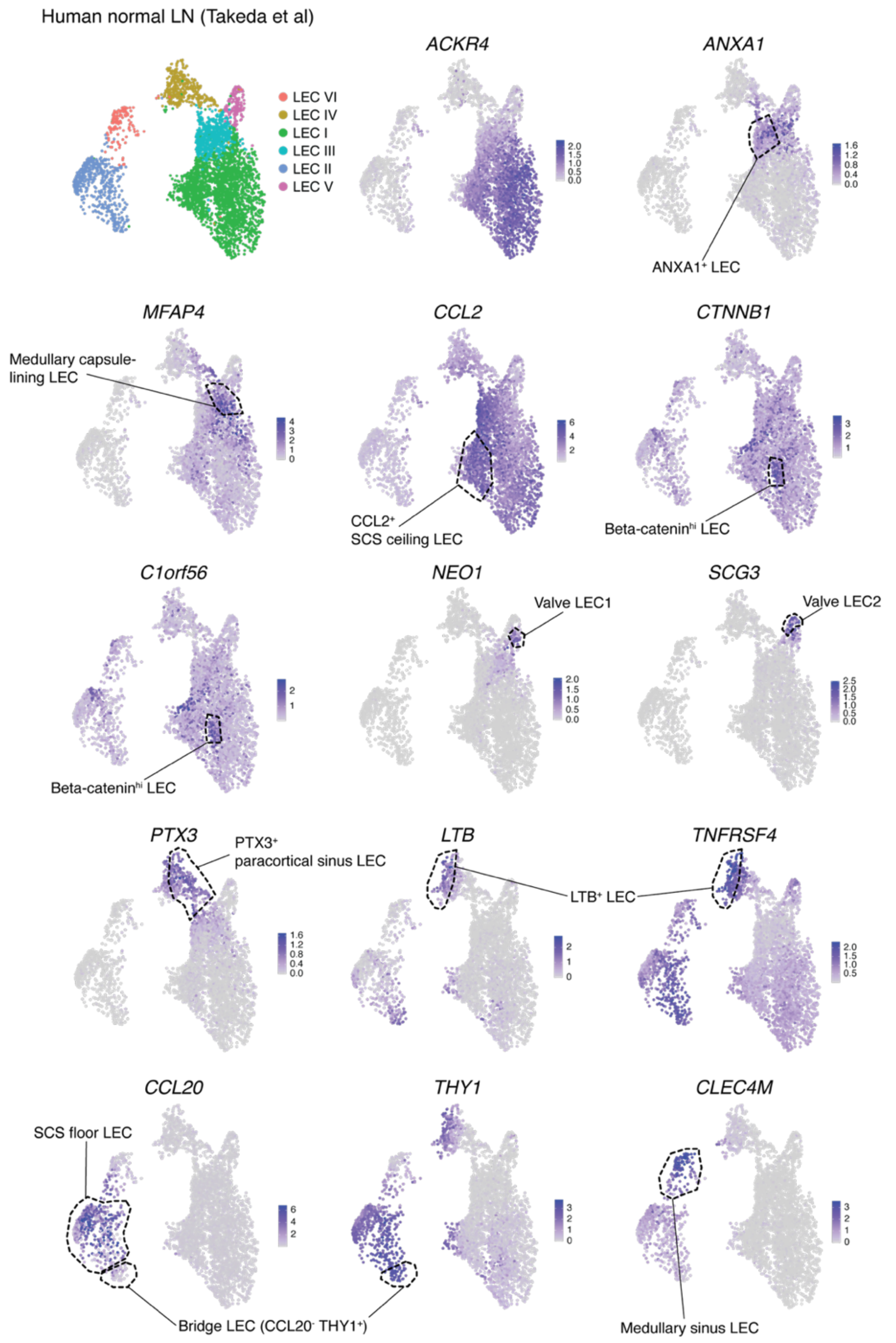

Supplementary Fig. 3 | Validation of newly identified LEC subsets in a published dataset. Reanalysis of our previously published human LN LEC scRNA-seq dataset (Takeda A et al., Immunity, 2019) to validate the newly identified LEC subsets. *ANXA1*$^{+}$ and *MFAP4*$^{+}$ medullary capsule-lining LECs were previously categorized as LEC III. *CCL2*$^{+}$ SCS ceiling LECs and *CTNNB1*$^{+}$ *C1orf56*$^{+}$ beta-catenin^{hi} LECs are found within the LEC I fraction. *PTX3*$^{+}$ paracortical sinus LECs and *LTB*$^{+}$ *TNFRSF4*$^{+}$ LECs are within LEC IV. *CCL20*$^{+}$ SCS floor LECs and *CCL20*$^{-}$*THY1*$^{+}$ bridge LECs are within the LEC II fraction. A head and neck LN (HNLN1) was used for this analysis.

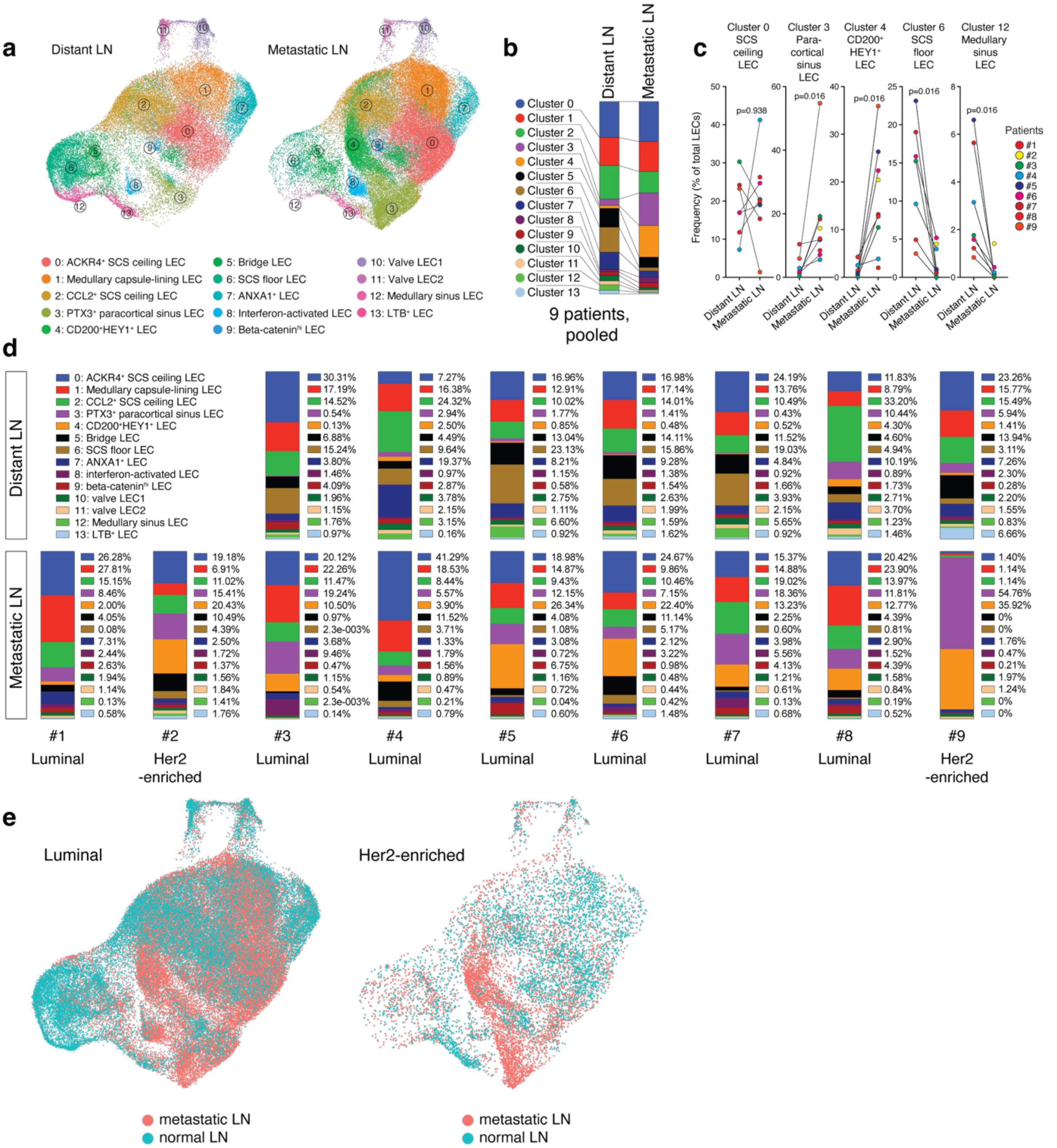

Supplementary Fig. 4 | LN LEC subsets in luminal and Her2-enriched breast cancer patients. a UMAP plots of LECs from distant LNs (left) and metastatic LNs (right), color-coded by cluster. Nine patients' samples were integrated for this analysis. **b, c** Frequencies of LEC subsets in distant and metastatic LNs, shown as pooled data of nine patients (**b**) and by individual patient (**c**) (two-tailed paired Student's *t*-test). Source data are provided as a Source Data file. **d** Frequencies of each LEC subset in individual patients. **e** UMAP plots of LN LECs from luminal (left) and HER2-enriched (right) breast cancer patients.

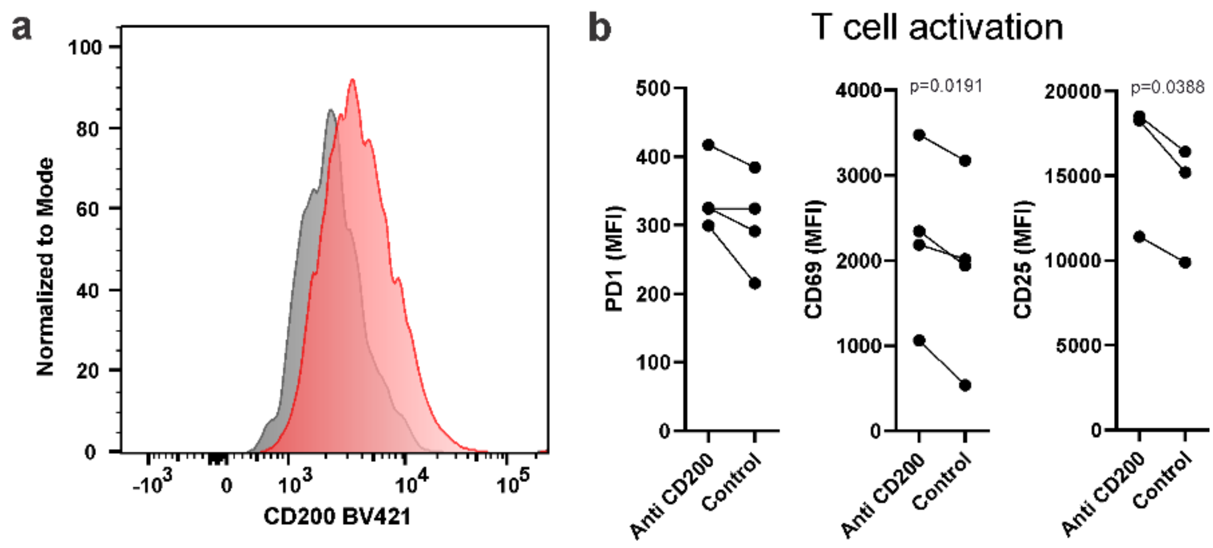

Supplementary Fig. 5 | T cell activation in the presence of CD200-expressing LECs

a A representative histogram showing CD200 expression (red) compared to isotype control (grey) on HLECs. **b** Expression of T cell activation markers CD25, CD69 and PD-1 on T cells after coculture with HLECs. HLECs had been treated with anti-CD200 or a control antibody before T cell were added and activated with CD3/CD28 Dynabeads. Data are shown as Tukey box plots (n=3-4, T cells from 3-4 healthy donors) and were analyzed with a two-tailed, paired Student's t-test. Source data, non-significant p-values and detailed n-numbers (donors) are provided in the Source Data file.

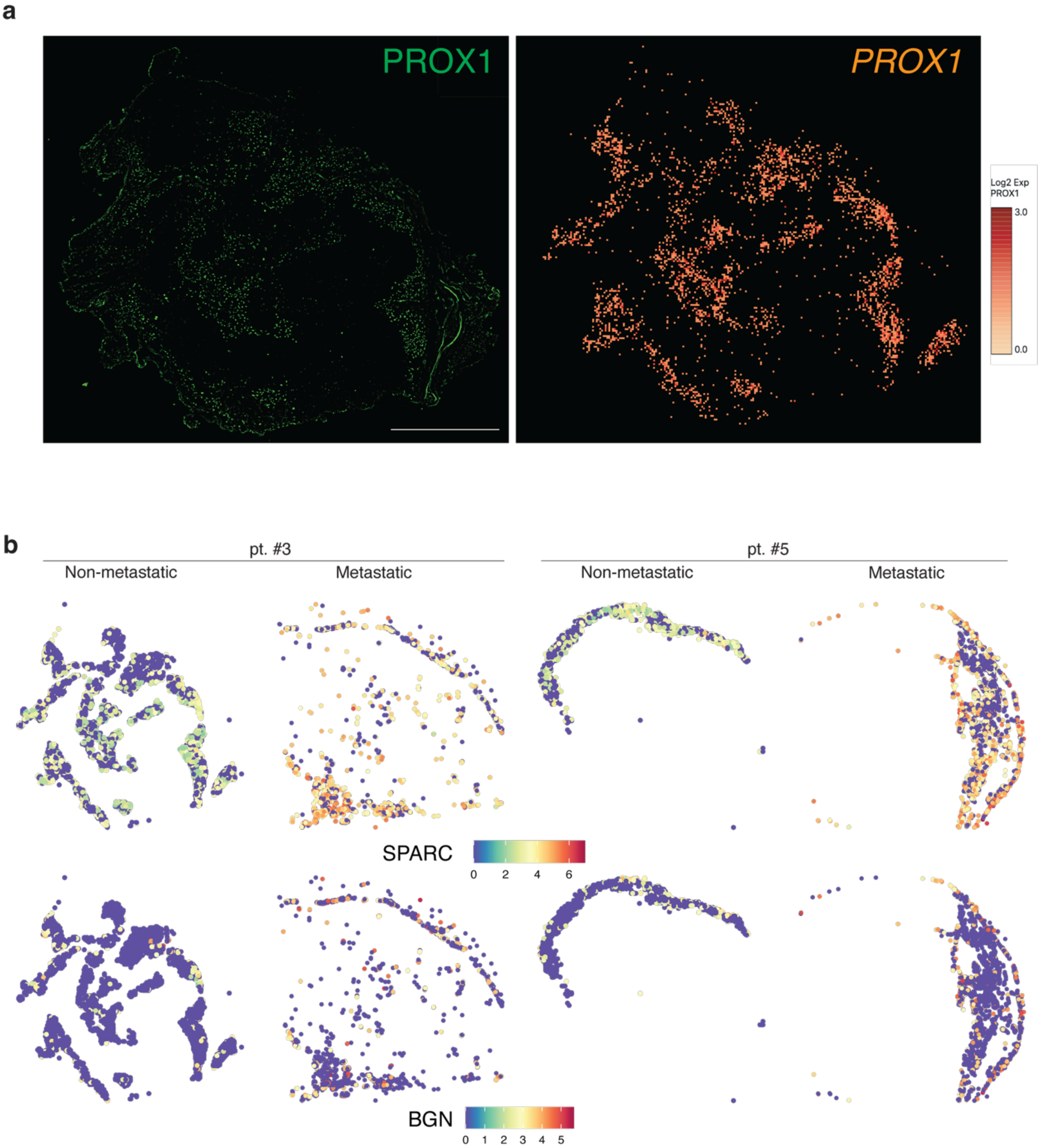

Supplementary Fig. 6 | Detection of LN LECs using high-resolution spatial transcriptomics Visium HD. a PROX1 expression at protein (left) and mRNA (right) level in human LNs. Scale bar = 500 µm. **b** *SPARC* and *BGN* expression in LECs of non-metastatic and metastatic LNs. Patient #3 and #5 were used for spatial transcriptomics analysis.

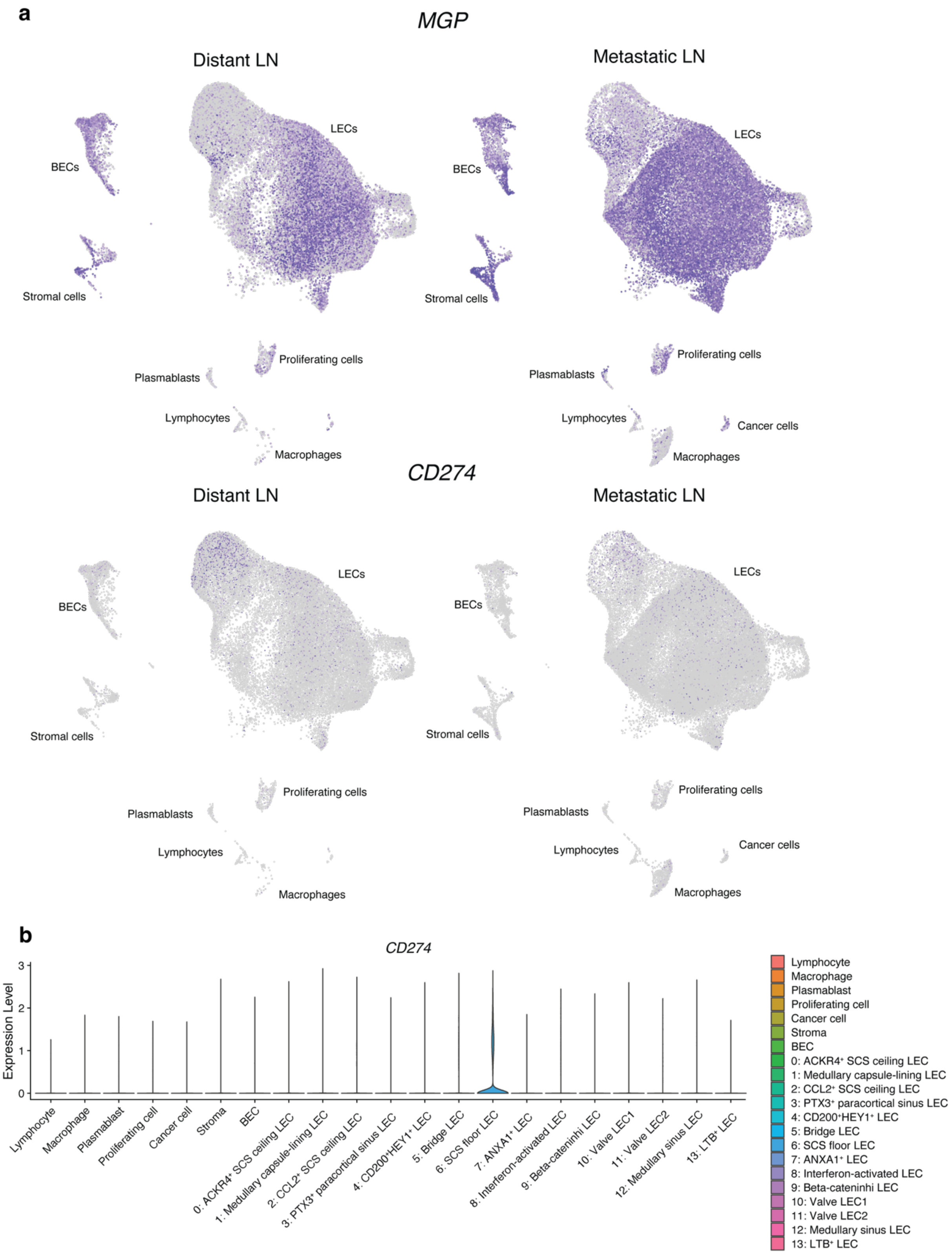

Supplementary Fig. 7 | MGP and CD274 (PD-L1) expression in distant and metastatic LNs. a Feature plots showing *MGP* and *CD274* expression in distant and metastatic LNs. *CD274* was downregulated in metastatic LNs. **b** Violin plot of *CD274* expression across all cell types in distant and metastatic LNs. Nine patients' samples were integrated for this analysis.

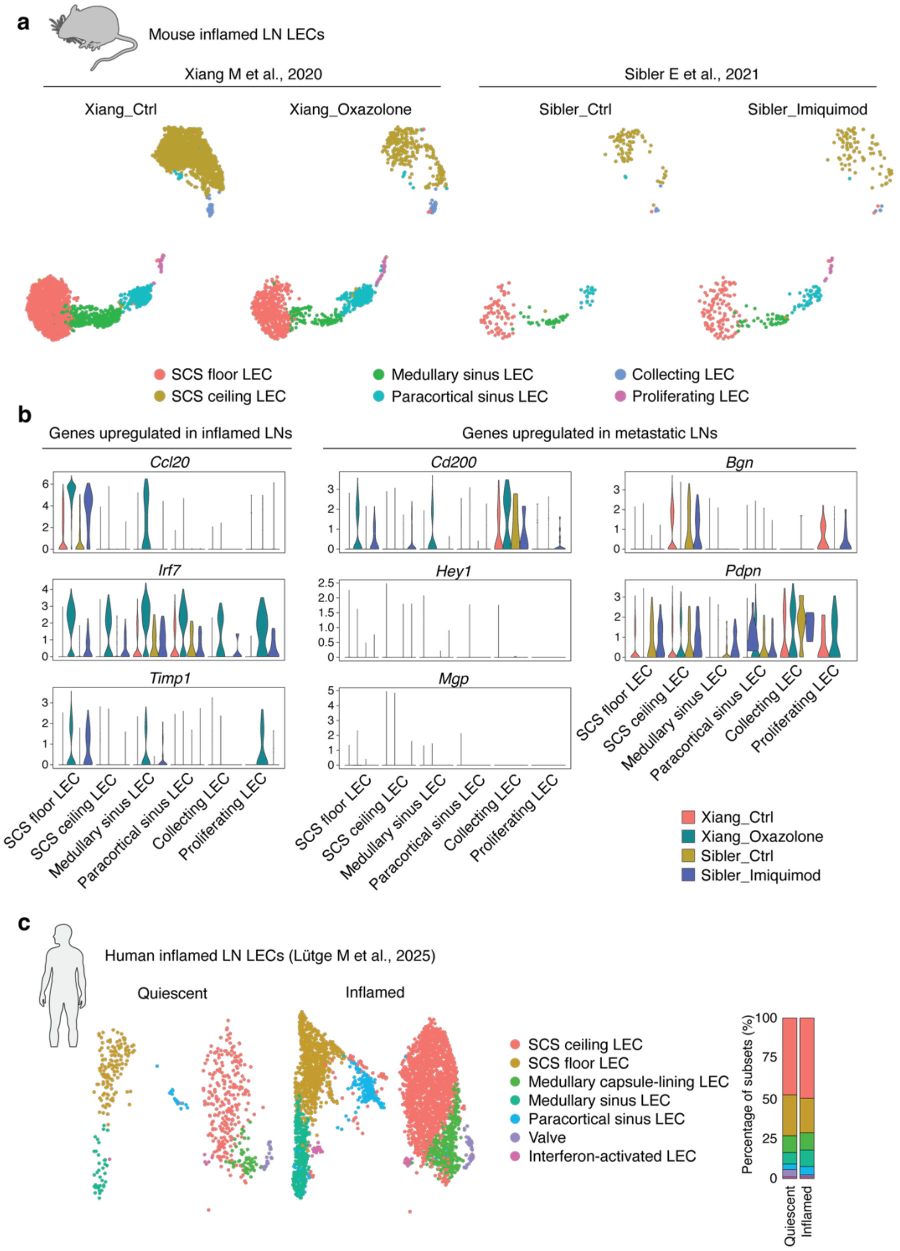

Supplementary Fig. 8 | LEC subsets in murine and human inflamed LNs. a, b Reanalysis of publicly available scRNA-seq datasets from murine inflamed LNs. The mice were immunized with either oxazolone or imiquimod (Xiang M et al., 2020; Sibler E et al., 2021). LEC cluster composition across treatments (a) and expression of genes upregulated in inflamed LN LECs and those upregulated in human metastatic LNs (b). **c** Reanalysis of a publicly available scRNA-seq dataset of human inflamed LNs (Lütge M et al., 2025). LEC clusters from quiescent (n=5 patients) and inflamed LNs (n=5 patients) are annotated based on our classification in Fig. 1 (left). The percentage of each LEC subset among total LECs is shown (right). Detailed information of samples is described in original publications. Illustrations are from NIAID NIH BioArt Source.

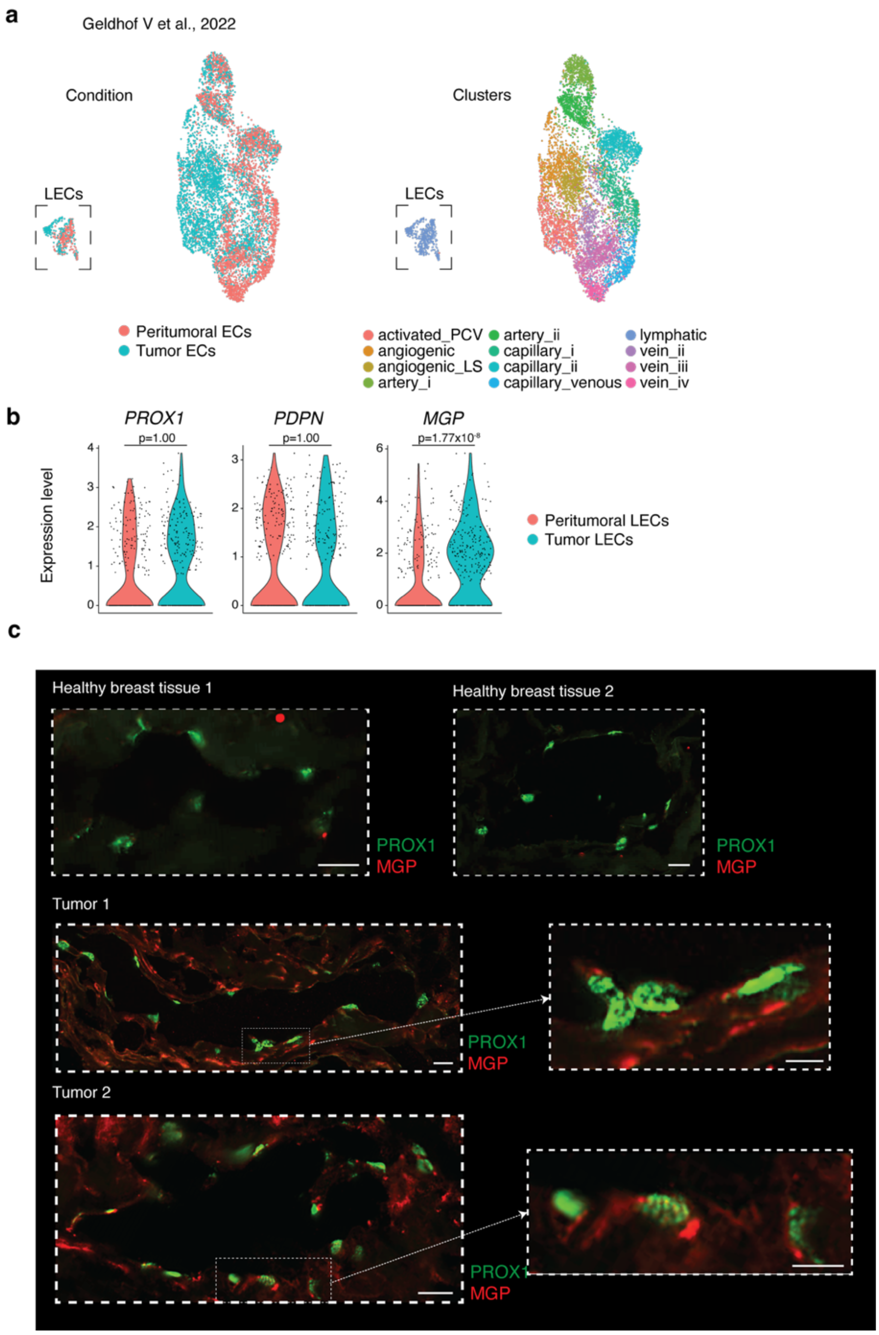

Supplementary Fig. 9 | MGP expression in lymphatics within primary tumor. a, b Reanalysis of publicly available scRNA-seq data of ECs from primary breast tumors (Geldhof V et al, Nat Comm, 2022). Plots are colored by condition (a, left; tumor EC n=8 patients; peri-tumoral EC n=7 patients), indicating the origin of ECs, and by EC subsets (a, right), annotated according to Geldhof et al.'s classification (a, right). The expression of PROX1, PDPN, and MGP in LECs from tumor and peritumoral regions (b). **c** Representative immunohistochemistry images showing MGP expression in the lymphatics of intact human breast tissue (n=3) and breast tumor tissue (n=2). Green, PROX1; red, MGP. Scale bar = 20 μm.

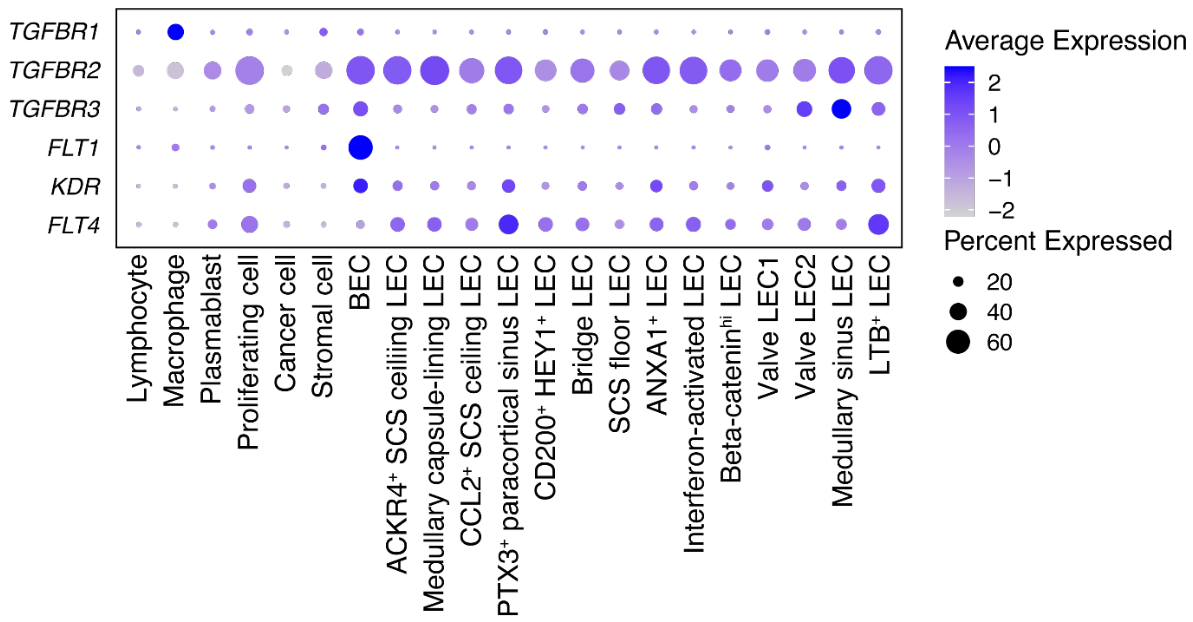

Supplementary Fig. 10 | Expression of receptors for TGF-β and VEGF in human LN cell subsets. Dot plots showing the expression of TGF-β receptors and VEGF receptors in immune cells, and stromal and EC subsets in human LNs. FLT1, KDR, FLT4 are also known as VEGFR1, VEGFR2, and VEGFR3, respectively. Nine patients' samples were integrated for this analysis.

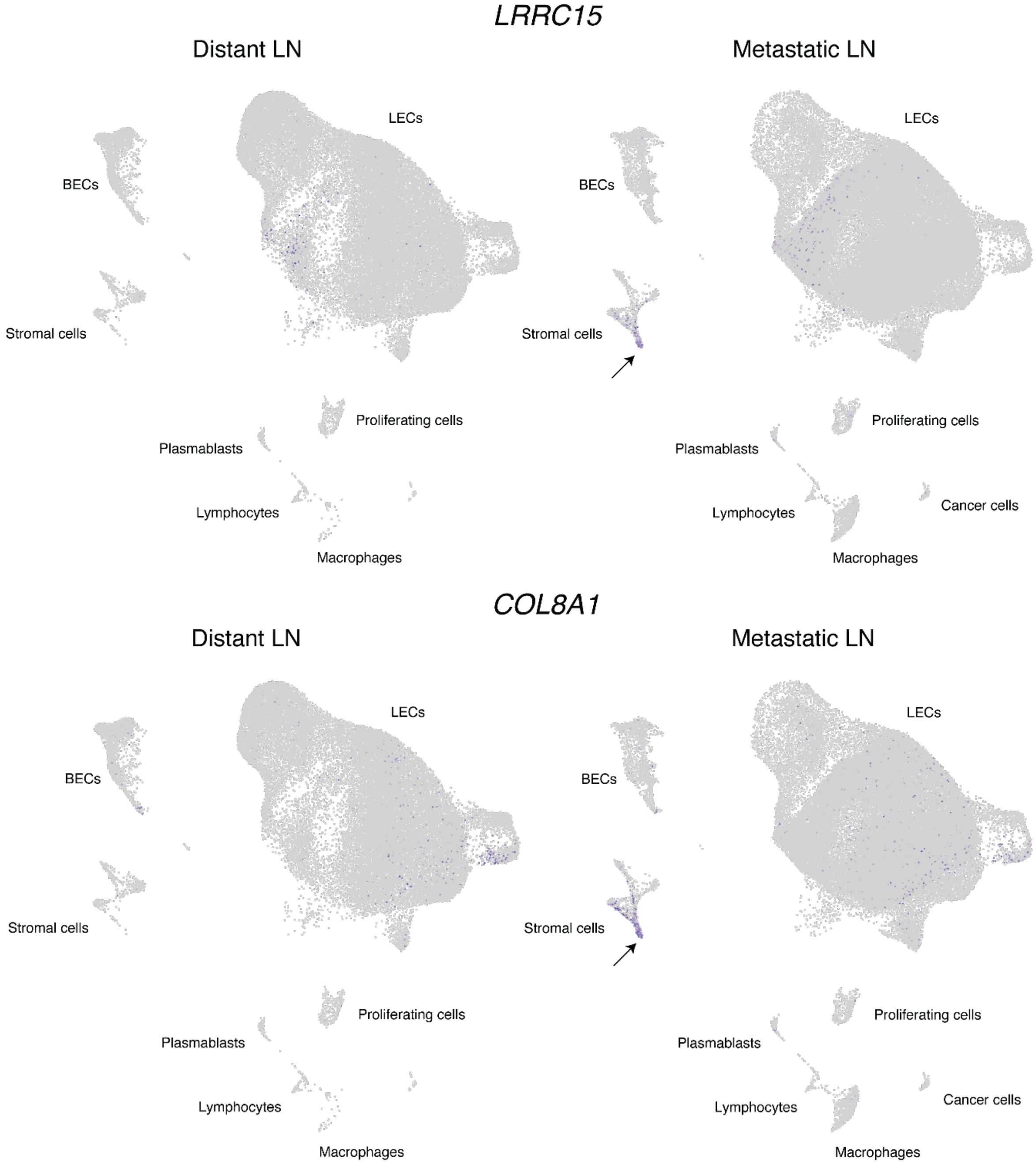

Supplementary Fig. 11 | TGF-β-Dependent LRRC15⁺ Cancer-Associated Fibroblasts in Metastatic LNs. LRRC15$^+$ cancer-associated fibroblasts (indicated by arrows) are present in metastatic LNs but not in distant LNs. Feature plots display markers of LRRC15$^+$ cancer-associated fibroblasts, including *LRRC15* and *COL8A1*. Nine patients' samples were integrated for this analysis.

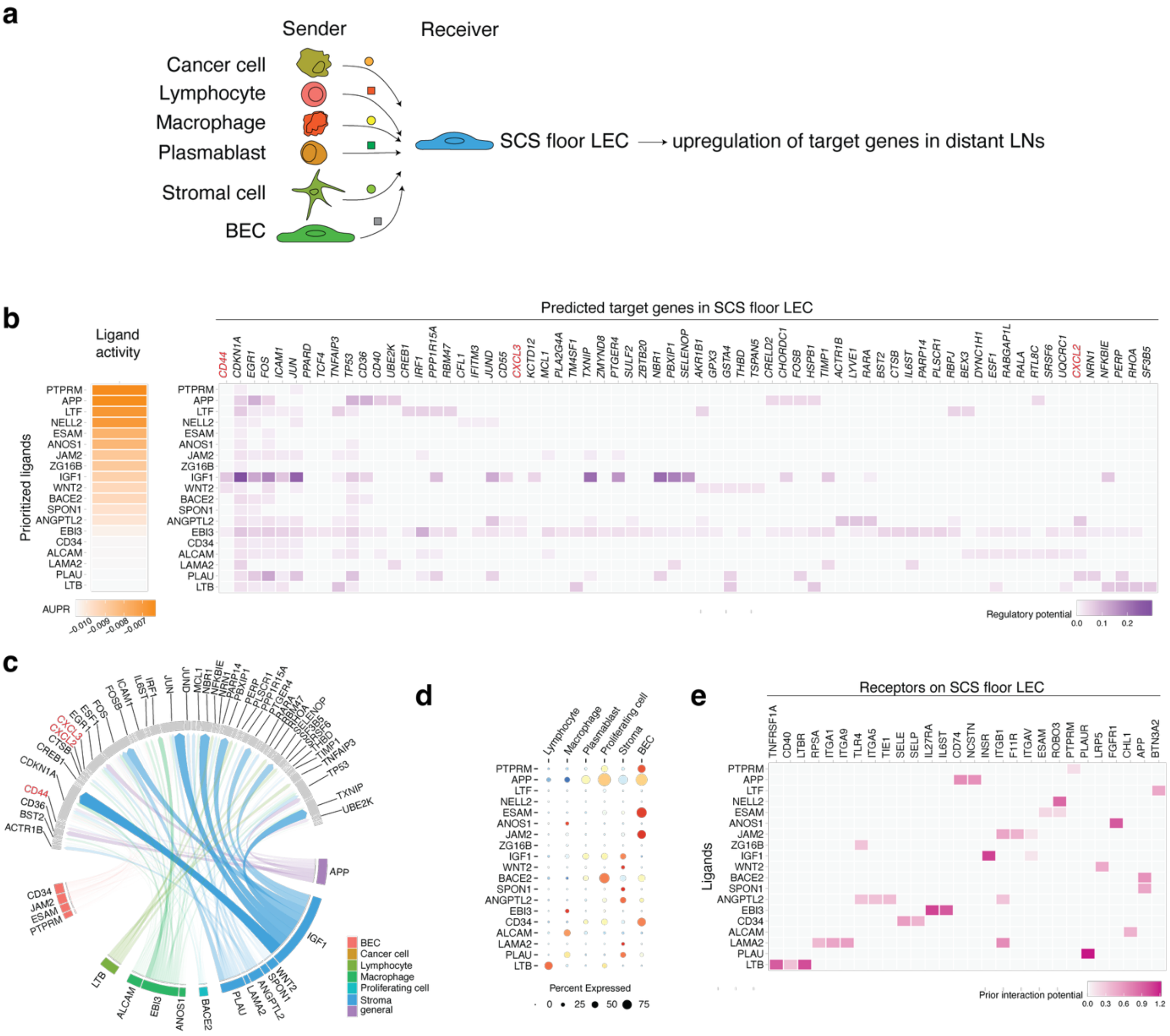

Supplementary Fig. 12 | NicheNet Intercellular Communication Analysis of SCS Floor LECs. a Schematic illustration of the NicheNet analysis to investigate the mechanisms maintaining SCS floor LECs. SCS floor LECs are predominantly found in distant LNs, and genes upregulated in these LNs were considered as crucial for the maintenance of this LEC type. SCS floor LECs were designated as the receiver, while other LN cell types were set as senders. **b** Predicted top ligands and their target genes in SCS floor LECs. **c** Circos plot showing connections between predicted ligands from LN cells and their potential target genes in SCS floor LECs. **d** Dot plot illustrating the expression of ligands in LN cells. **e** Potential receptors expressed by SCS floor LECs associated with each predicted ligand. Nine patients' samples were integrated for this analysis.

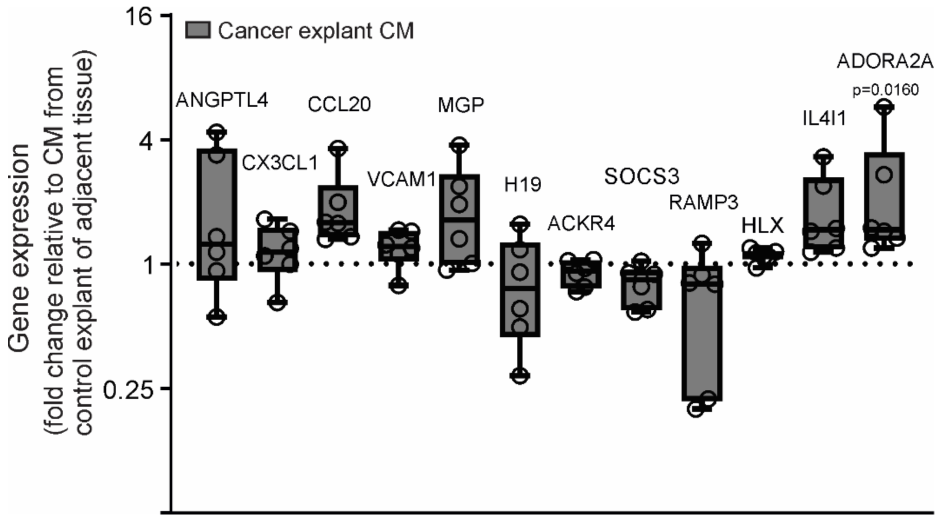

Supplementary Fig. 13 | Gene expression in HLECs after CM exposure. Changes in CM-exposed LECs are shown as determined by qPCR. CM from three cancer explants were used and compared to CM from corresponding explants of adjacent tissue with two different types of LECs (n = 6; HLECs from 2 different sources exposed to cancer explant CM from 3 donors). Data were analyzed with 2-way ANOVA and Sidak's multiple comparison test. Data are depicted as Tukey box plots. Source data, non-significant p-values and detailed n-numbers are provided in the Source Data file.

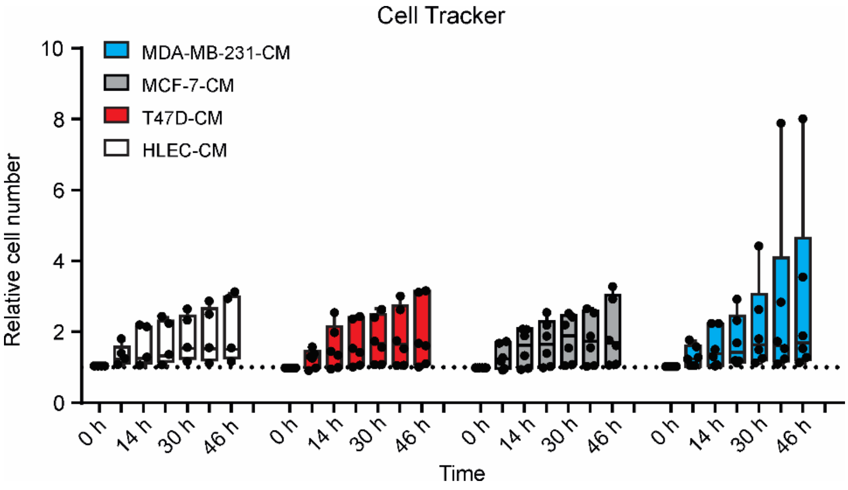

Supplementary Fig. 14 | LEC proliferation in the presence of cancer cell CM. The proliferation of LECs exposed to different CM is shown. LECs were labelled with 6.5 µM CellTracker Red, exposed to different CM and their relative cell number over the course of two days was determined at different timepoints. Data are shown as Tukey box plots (n=5-6; MDA-MD-231, MCF-7, T47D and HLEC cells), analyzed with 2-way ANOVA (mixed effects) with Sidak correction. The center line of the box plots represents the median, the box the 25th to 75th percentiles and the whiskers inner fences. Source data, non-significant p-values and detailed experiment and n-numbers (biologically independent samples of cultured cells) are provided in the Source Data file.

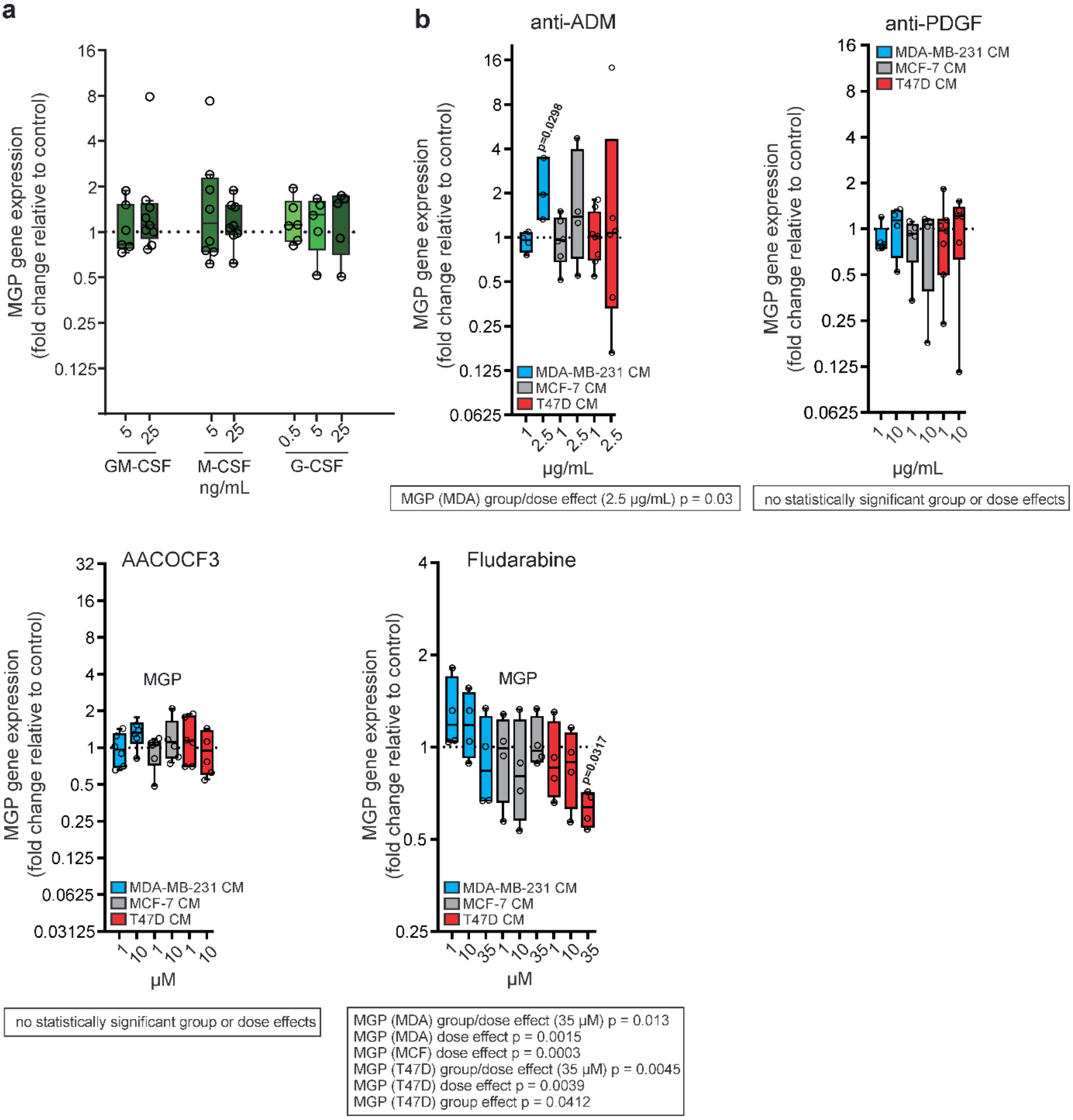

Supplementary Fig. 15 | Gene expression of MGP after recombinant cytokine and antibody exposure. a Gene expression of MGP in LECs after direct exposure to recombinant GM-CSF (5 or 25 ng/mL, n=7-9 HLECs), M-CSF (5 or 25 ng/mL, n=8 HLECs) or G-CSF (0.5, 5 or 25 ng/mL, n=5-6 HLECs), respectively are shown. Data are depicted as Tukey box plots, analyzed with 2-way ANOVA (mixed model) with Sidak correction. **b** Gene expression of MGP in LECs are shown following their exposure to different modified CM. CM were generated in the presence of 1 and 2.5 µg/mL anti-ADM antibody (n = 4-8 HLECs), 1 and 10 µg/mL anti-PDGF antibody (n = 4-7 HLECs), 1 and 10 µM AACOCF3 (n = 6 HLECs) and 1, 10 and 35 µM Fludarabine (n = 4 HLECs) or their control antibody/substance, respectively. Data are shown as Tukey box plots (n=4-8). Data were analyzed with a linear mixed model. The center

line of the box plots represents the median, the box the 25th to 75th percentiles and the whiskers inner fences. Statistics of group and dose effects are presented within the boxes; significant differences in comparison to the controls (defined as 1) are indicated by p value. Source data, non-significant p-values and detailed experiment and n-numbers (biologically independent samples of cultured cells) are provided in the Source Data file.

.

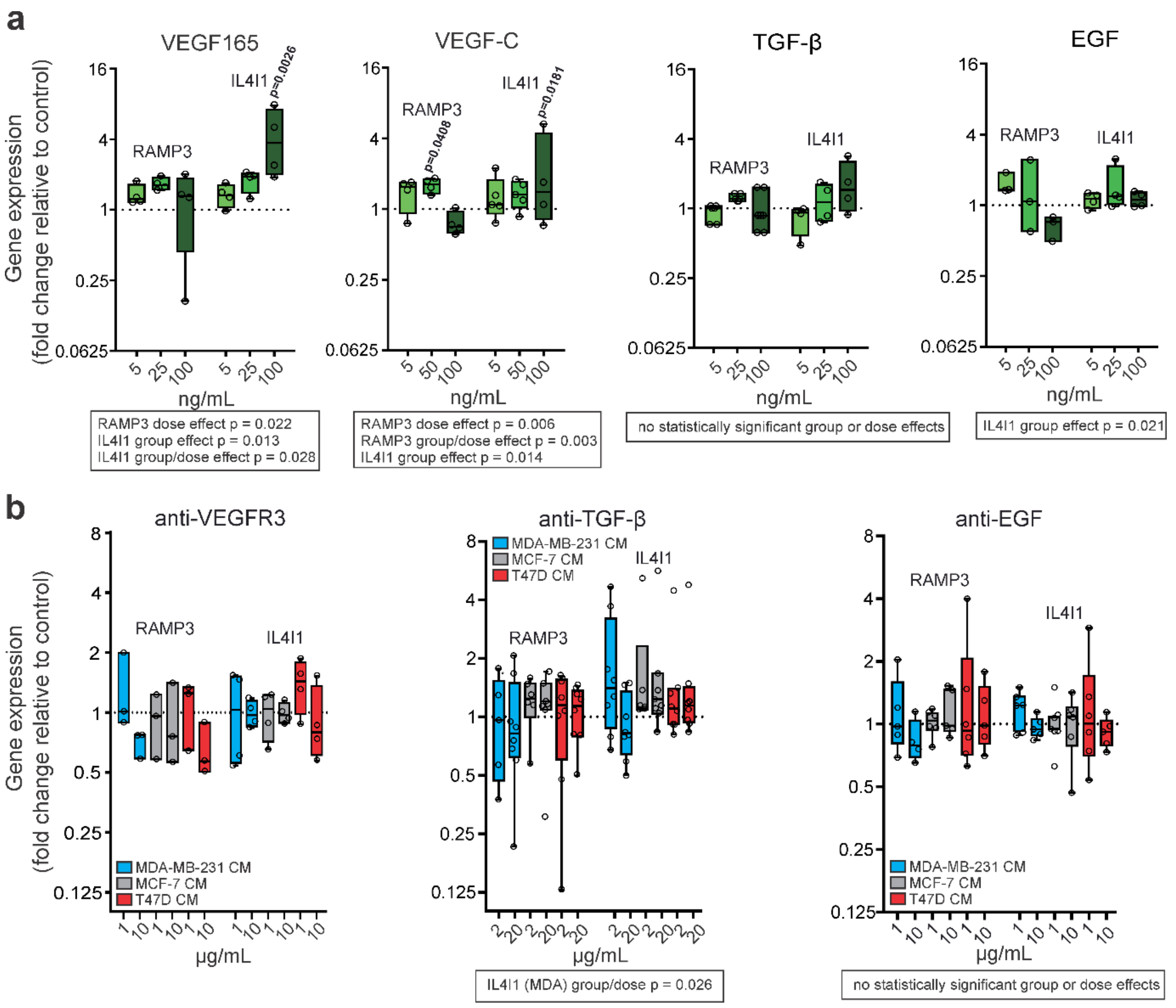

Supplementary Fig. 16 | Gene expression of RAMP3 and IL4I1 after recombinant cytokine or antibody exposure. a

Expression of *RAMP3* and *IL4I1* in LECs after direct exposure to recombinant VEGF165 (5, 25, or 100 ng/mL) (n = 4 HLECs), VEGF-C (5, 50, or 100 ng/mL) (n = 4 HLECs), TGF-β (5, 25, or 100 ng/mL) (n = 3 HLECs), or EGF (5, 25, or 100 ng/mL) (n = 3 HLECs) are shown. Data are depicted as Tukey box plots and analyzed using linear mixed models fitted separately for each parameter with group (recombinant vs control) and dose and their interaction as fixed effects. **b** Gene expression changes in modified CM-exposed LECs are shown as determined by qPCR. CM was generated in the presence of antibodies against VEGFR3 (1 or 10 µg/mL, n = 3–4 HLECs), TGF-β (2 or 20 µg/mL, n = 5–10 HLECs), and EGF (1 or 10 µg/mL, n = 3–5 HLECs), compared to Isotype control exposed samples and data are depicted as Tukey box plots showing relative gene changes and analyzed using linear mixed models fitted separately for each parameter with group (antibody vs control) and dose and their interaction as fixed

effects. Source data, non-significant p-values and detailed experiment and n-numbers (biologically independent samples of cultured cells) are provided in the Source Data file.

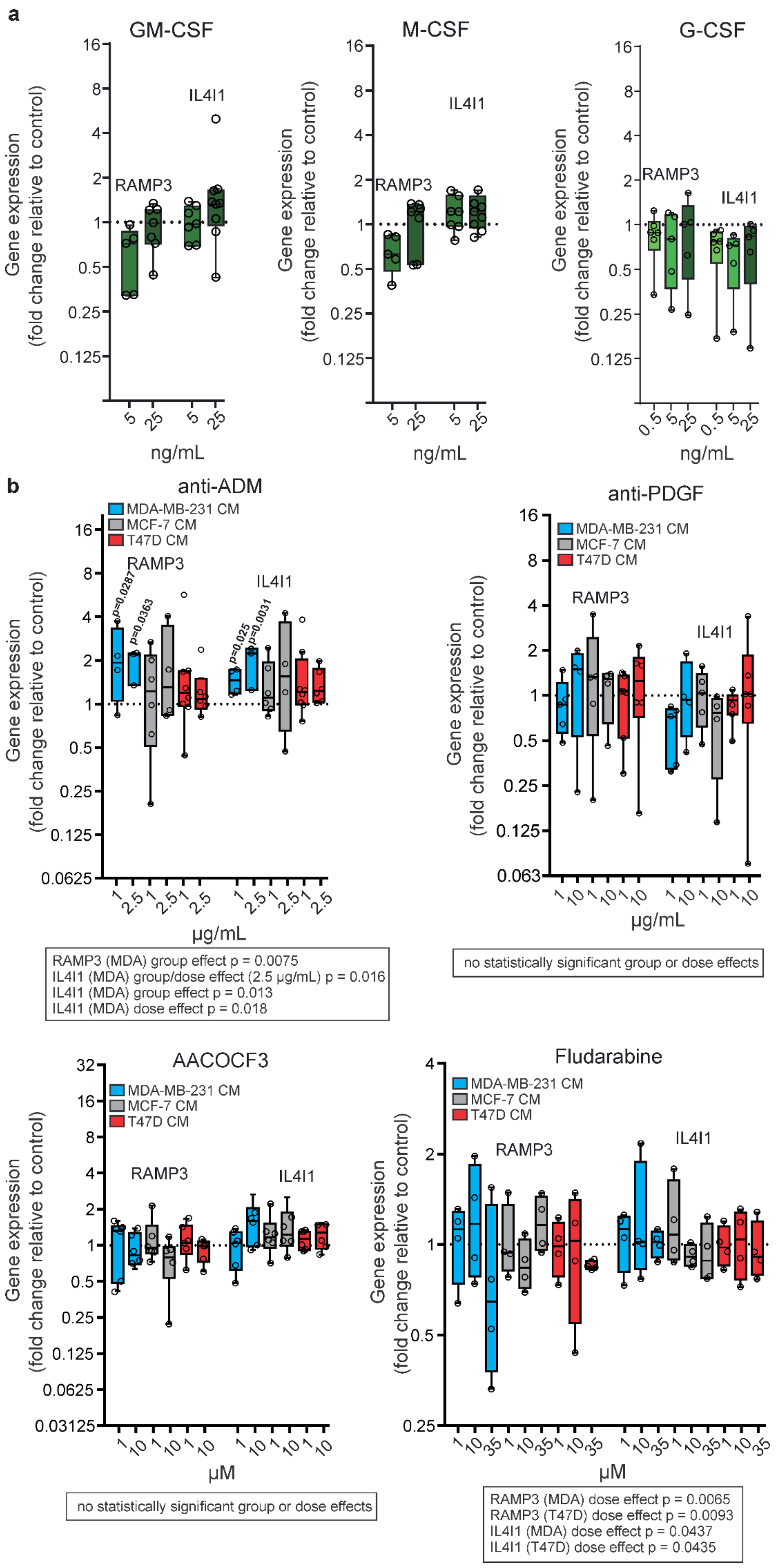
a
GM-CSF
M-CSF
G-CSF
Gene expression
(fold change relative to control)
RAMP3
IL4I1
ng/mL
b
anti-ADM
anti-PDGF
AACOCF3
Fludarabine
MDA-MB-231 CM
MCF-7 CM
T47D CM
p=0.0287
p=0.0363
p=0.025
p=0.0031
µg/mL
µM
RAMP3 (MDA) group effect p = 0.0075
IL4I1 (MDA) group/dose effect (2.5 µg/mL) p = 0.016
IL4I1 (MDA) group effect p = 0.013
IL4I1 (MDA) dose effect p = 0.018
no statistically significant group or dose effects
no statistically significant group or dose effects
RAMP3 (MDA) dose effect p = 0.0065
RAMP3 (T47D) dose effect p = 0.0093
IL4I1 (MDA) dose effect p = 0.0437
IL4I1 (T47D) dose effect p = 0.0435

Supplementary Fig. 17 | Gene expression of RAMP3 and IL4I1 after recombinant cytokine or antibody exposure

a Gene expression of RAMP3 and IL4I1 in LECs after direct exposure to recombinant GM-CSF (5 or 25 ng/mL, n = 5-7 HLECs), M-CSF (5 or 25 ng/mL, n = 5-9 HLECs) or G-CSF (0.5, 5 or 25 ng/mL, n = 5-6 HLECs), respectively are shown. Data are depicted as Tukey box plots, analyzed with a one-way ANOVA (mixed model) with Sidak correction. **b** Gene expression of RAMP3 and IL4I1 in LECs are shown following their exposure to different modified CM. CM were generated in the presence of 1 and 2.5 µg/mL anti-ADM antibody (n = 5-8 HLECs), 1 and 10 µg/mL anti-PDGF antibody (n = 4-7 HLECs), 1 and 10 µM AACOCF3 (n = 6 HLECs) and 1, 10 and 35 µM Fludarabine (n = 4 HLECs) or their control antibody/substance, respectively. Data are shown as Tukey box plots. Data were analyzed with one-way ANOVA and a linear mixed model. The center line of the box plots represents the median, the box the 25th to 75th percentiles and the whiskers inner fences. Source data, non-significant p-values and detailed experiment and n-numbers (biologically independent samples of cultured cells) are provided in the Source Data file.

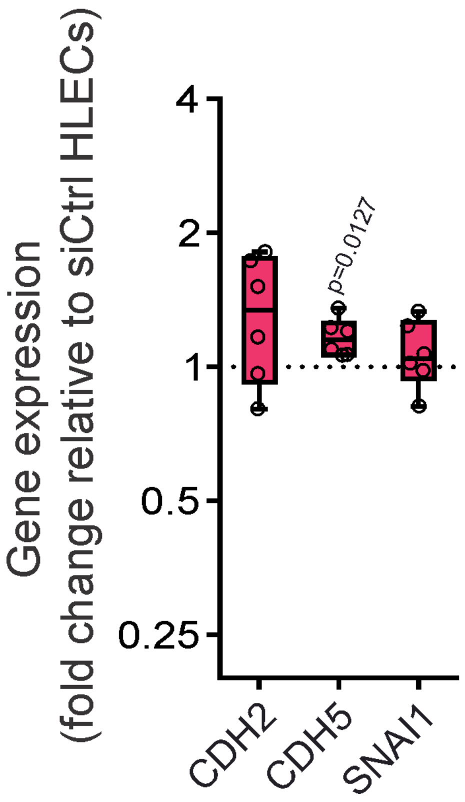

Supplementary Fig. 18 | siMGP effect on EndMT.

Gene expression of CDH2, CDH5 and SNAIl in MGP silenced LECs are shown as Tukey box plots (n=6 HLECs) and analyzed with the two-sided Wilcoxon matched-pairs signed rank test. The center line of the box plots represents the median, the box the 25th to 75th percentiles and the whiskers inner fences. Source data, non-significant p-values and detailed experiment and n-numbers (biologically independent samples of cultured cells) are provided in the Source Data file.
