# Supplementary material for: Breast cancer remodels lymphatics in sentinel lymph nodes
Source: Nat Commun. 2025 Nov 17;16:10056. doi: 10.1038/s41467-025-64981-z (PMC12623973; doi:10.1038/s41467-025-64981-z)
Supplement: Supplementary file 2 — Description of Addtional Supplementary File [file 41467_2025_64981_MOESM2_ESM.pdf]

Description of Additional Supplementary File

Supplementary Data 1. Up and down regulated genes by LN metastasis in different clusters. DEGs were identified using the Wilcoxon Rank Sum test.
