# Supplementary material for: Breast cancer remodels lymphatics in sentinel lymph nodes
Source: Nat Commun. 2025 Nov 17;16:10056. doi: 10.1038/s41467-025-64981-z (PMC12623973; doi:10.1038/s41467-025-64981-z)
Supplement: Supplementary file 4 — Reporting Summary [file 41467_2025_64981_MOESM4_ESM.pdf]

nature portfolio

Corresponding author(s): Akira Takeda and Sirpa Jalkanen

Last updated by author(s): Sep 1, 2025

Reporting Summary

Nature Portfolio wishes to improve the reproducibility of the work that we publish. This form provides structure for consistency and transparency in reporting. For further information on Nature Portfolio policies, see our Editorial Policies and the Editorial Policy Checklist.

Statistics

For all statistical analyses, confirm that the following items are present in the figure legend, table legend, main text, or Methods section.

n/a	Confirmed	
☐	☒	The exact sample size (n) for each experimental group/condition, given as a discrete number and unit of measurement
☐	☒	A statement on whether measurements were taken from distinct samples or whether the same sample was measured repeatedly
☐	☒	The statistical test(s) used AND whether they are one- or two-sided<br>*Only common tests should be described solely by name; describe more complex techniques in the Methods section.*
☒	☐	A description of all covariates tested
☐	☒	A description of any assumptions or corrections, such as tests of normality and adjustment for multiple comparisons
☐	☒	A full description of the statistical parameters including central tendency (e.g. means) or other basic estimates (e.g. regression coefficient) AND variation (e.g. standard deviation) or associated estimates of uncertainty (e.g. confidence intervals)
☐	☒	For null hypothesis testing, the test statistic (e.g. F, t, r) with confidence intervals, effect sizes, degrees of freedom and P value noted<br>*Give P values as exact values whenever suitable.*
☒	☐	For Bayesian analysis, information on the choice of priors and Markov chain Monte Carlo settings
☒	☐	For hierarchical and complex designs, identification of the appropriate level for tests and full reporting of outcomes
☒	☐	Estimates of effect sizes (e.g. Cohen's d, Pearson's r), indicating how they were calculated

Our web collection on statistics for biologists contains articles on many of the points above.

Software and code

Policy information about availability of computer code

Data collection	For cell sorting, Sony SH800S software was used; for acquisition of cytometry data, BD FACS Diva was used; GEX libraries were analyzed using Cell Ranger 3.0.1 or 3.1.0 (10x Genomics);For image acquisition, ZEN 2.3 SP1 black edition and LAX X LS were used. For qPCR, the software of the QuantStudio3 was used; For VisiumHD analysis, Space Ranger HD 3.1.3 (10x Genomics) was used.
Data analysis	FACS files were analyzed using FlowJo 10.10 (BD Bioscience); ScRNA-seq data was analyzed using Seurat v4 on R4.3.2. Microscopic images were analyzed using ImageJ 2.14.0. Spatial trancriptomics data was analyzed using Seurat v5.2.0. Statistical analyses were performed using GraphPad Prism 10 (Graphpad). Detailed descriptions can be found in Materials and Methods section. Code iss available in GitLab at https://gitlab.utu.fi/akitak/lymphatics-in-cancer.

For manuscripts utilizing custom algorithms or software that are central to the research but not yet described in published literature, software must be made available to editors and reviewers. We strongly encourage code deposition in a community repository (e.g. GitHub). See the Nature Portfolio guidelines for submitting code & software for further information.

Data

Policy information about availability of data

All manuscripts must include a data availability statement. This statement should provide the following information, where applicable:

- Accession codes, unique identifiers, or web links for publicly available datasets
- A description of any restrictions on data availability
- For clinical datasets or third party data, please ensure that the statement adheres to our policy

ScRNA-seq , bulk RNA-seq and VisiumHD data supporting the results of this study have been deposited in the Gene Expression Omnibus (GEO) with the accession numbers GSE248214, GSE248076, and GSE298872 respectively.

Research involving human participants, their data, or biological material

Policy information about studies with human participants or human data. See also policy information about sex, gender (identity/presentation), and sexual orientation and race, ethnicity and racism.

Reporting on sex and gender

For scRNAseq, paired metastatic and non-metastatic lymph nodes (LNs) were collected from 9 females with breast cancer undergoing mastectomy and axillary LN removal. For T cell stimulation assay, T cells were extracted from a total of 4 healthy volunteers, 2 males and 2 females.

Reporting on race, ethnicity, or other socially relevant groupings

Please specify the socially constructed or socially relevant categorization variable(s) used in your manuscript and explain why they were used. Please note that such variables should not be used as proxies for other socially constructed/relevant variables (for example, race or ethnicity should not be used as a proxy for socioeconomic status).

Provide clear definitions of the relevant terms used, how they were provided (by the participants/respondents, the researchers, or third parties), and the method(s) used to classify people into the different categories (e.g. self-report, census or administrative data, social media data, etc.)

Please provide details about how you controlled for confounding variables in your analyses.

Population characteristics

Samples were collected from patients who had not undergone any prior treatment. The median age of patients is 70. T cells were obtained from healthy volunteers with a median age of 35.

Recruitment

Human samples were obtained from research volunteers of Turku University Hospital. Written informed consent was obtained from all patients. Similarly, human T cells were obtained from blood donations by healthy volunteers working in the MediCity Research Laboratories after written informed consent was obtained.

Ethics oversight

The sample collection was conducted under the license EMTK: 132/2016. The samples were used with the permission of the Institutional Review Board of Medicolegal Affairs (Helsinki and Turku, Finland). Samples from blood donors were anonymized during the cell separation and no other information was collected from the donors. No approval from the local ethics committee was obtained as anonymous blood sample collection and analysis is not considered to be regulated by Finnland's Medical Research Act No. 499/1999.

Note that full information on the approval of the study protocol must also be provided in the manuscript.

Field-specific reporting

Please select the one below that is the best fit for your research. If you are not sure, read the appropriate sections before making your selection.

[x] Life sciences [ ] Behavioural & social sciences [ ] Ecological, evolutionary & environmental sciences

For a reference copy of the document with all sections, see nature.com/documents/nr-reporting-summary-flat.pdf

Life sciences study design

All studies must disclose on these points even when the disclosure is negative.

Sample size

Human patient sample sizes were determined by the availability of surgically resected patient samples. Although no power calculation was performed, the number of biological replicates was sufficient to observe reproducible results across independent patients. For functional assays, sample sizes were chosen based on prior experience in the field, where similar numbers have been adequate to demonstrate significant effects.

Data exclusions

No samples were excluded.

Replication

All experiments were repeated for at least two or three times for reproducibility of the findings. ScRNA-seq experiments were conducted nine times, utilizing freshly available human samples.

Randomization

Sample allocation into groups was not randomized. For patient samples, samples were assigned to groups based on their clinical and biological characteristics (e.g., metastatic vs. non-metastatic lymph nodes). As these group identities were predetermined by pathology, random allocation was not relevant to the study. For in vitro assays, samples were allocated according to the experimental conditions. Thus, allocation was predefined by study design.

Blinding	Blinding was not done because the study involved pre-defined human patient samples where group identify was inherently evident.

Reporting for specific materials, systems and methods

We require information from authors about some types of materials, experimental systems and methods used in many studies. Here, indicate whether each material, system or method listed is relevant to your study. If you are not sure if a list item applies to your research, read the appropriate section before selecting a response.

Materials & experimental systems

n/a	Involved in the study
☐	☒ Antibodies
☐	☒ Eukaryotic cell lines
☒	☐ Palaeontology and archaeology
☒	☐ Animals and other organisms
☒	☐ Clinical data
☒	☐ Dual use research of concern
☒	☐ Plants

Methods

n/a	Involved in the study
☒	☐ ChIP-seq
☐	☒ Flow cytometry
☒	☐ MRI-based neuroimaging

Antibodies

Antibodies used	AF488 anti-pan-cytokeratin, ThermoFisher Scientific, MA5-18156 (1:100); PE anti-PDPN, Biolegend, 337004 (1:100); AF488 anti-CD45, Biolegend, 304019 (1:100); APC anti-CD31, Biolegend, 303115 (1:100); anti-PROX1, R&D Systems, AF2727 (1:100); anti-MARCO, Atlas Antibodies, HPA063793 (1:50); anti-MGP, Novus Biologicals, NBP2-45844 (1:150); BV421 anti-CD200, Biolegend, 329209 (1:50); mouse IgG2a control (553454), BD; anti-mouse IgG2a AF546 (A21133), Invitrogen; anti-adrenomedulin (AF6108) (1:80-1:200), R&D Systems; anti-EGF (AB-236-NA) (1:100-1:1000), R&D Systems; goat IgG control (AB-108-C), R&D Systems; anti-PDGF (AB-20-NA) (1:100-1:1000), R&D Systems; rabbit IgG control (AB-105-C), R&D Systems; anti-MGP (A5439) (1:100), Abclonal; anti-TGFb (BE0057) (2-20 µg/mL), BioXCell; mouse IgG1 control (BE0083), BioXCell; anti-VEGFR3 (IMC-3C5) (1-10 µg/mL), gift from Kari Alitalo but produced by Eli Lilly; human IgG1 control (BE0297), BioXCell. anti-MGP (10734-1-AP) (1:120), Proteintech; rabbit IgG control (30000-0-A), Proteintech; anti-CD200 blocking antibody (A2632), Seleckchem; Human IgG2sa Isotype control (A3176), Seleckchem; anti-His-PE antibody, Biolegend, 362603; anti-Podoplanin PE (337003) (1:50), Biolegend; anti-CD200 (ALXN6000) (5µg/mL), Seleckchem; anti-CD25 BV650 (563719) (1:50), BD; anti-CD69 BV609 (310937) (1:50), Biolegend; anti-CD279 FITC (557860) (1:50), BD.
Validation	PDPN, CD45, CD31, PROX1, and MARCO antibodies were validated in our previous publication (Takeda et al., Immunity, 2019). Other antibodies were validated by manufacturers.

Eukaryotic cell lines

Policy information about cell lines and Sex and Gender in Research

Cell line source(s)	MDA-MB-231 (ATCC); T47D (ATCC); MCF-7 (ATCC); PC3 (ATCC); HCC1954 (ATCC); HLECs from ScienCell (2500), CellBiologics (H-6092) and isolated from fresh LNs obtained from the Turku University hospital (donors were female).
Authentication	MDA-MB-231, T47D, MCF-7, PC3 and HCC1954 cells had been obtained from ATCC, maintained in-house and had been verified for a previous publication (Hollmén et al., Oncoimmunology 2015). The cells were used with low passage after that.
Mycoplasma contamination	All cell lines were tested negatively for mycoplasma
Commonly misidentified lines (See ICLAC register)	*Name any commonly misidentified cell lines used in the study and provide a rationale for their use.*

Plants

Seed stocks	*Report on the source of all seed stocks or other plant material used. If applicable, state the seed stock centre and catalogue number. If plant specimens were collected from the field, describe the collection location, date and sampling procedures.*
Novel plant genotypes	*Describe the methods by which all novel plant genotypes were produced. This includes those generated by transgenic approaches, gene editing, chemical/radiation-based mutagenesis and hybridization. For transgenic lines, describe the transformation method, the number of independent lines analyzed and the generation upon which experiments were performed. For gene-edited lines, describe the editor used, the endogenous sequence targeted for editing, the targeting guide RNA sequence (if applicable) and how the editor was applied.*
Authentication	*Describe any authentication procedures for each seed stock used or novel genotype generated. Describe any experiments used to assess the effect of a mutation and, where applicable, how potential secondary effects (e.g. second site T-DNA insertions, mosiacism, off-target gene editing) were examined.*

Flow Cytometry

Plots

Confirm that:

- [x] The axis labels state the marker and fluorochrome used (e.g. CD4-FITC).
- [x] The axis scales are clearly visible. Include numbers along axes only for bottom left plot of group (a 'group' is an analysis of identical markers).
- [x] All plots are contour plots with outliers or pseudocolor plots.
- [x] A numerical value for number of cells or percentage (with statistics) is provided.

Methodology

Sample preparation	Single cell suspension was created from LNs by enzymatic dissociation (described in methods).
Instrument	Sony SH800S for cell sorting; BD LSRFortessa for analysis.
Software	BD FACSDiva was used for data collection. Data was analyzed using FlowJo (BD Bioscience).
Cell population abundance	Purity of sorted LECs was evaluated by analysing scRNA-seq data.
Gating strategy	LN LECs were isolated by selecting for Live CD45- PDPN+ CD31+ cells through gating.

- [x] Tick this box to confirm that a figure exemplifying the gating strategy is provided in the Supplementary Information.
